# *Mycobacterium bovis* Bacillus Calmette-Guérin-Induced Macrophage Cytotoxicity against Bladder Cancer Cells

**DOI:** 10.1155/2010/357591

**Published:** 2010-09-01

**Authors:** Yi Luo, Matthew J. Knudson

**Affiliations:** Department of Urology, University of Iowa, 375 Newton Road, 3202 MERF, Iowa City, IA 52242, USA

## Abstract

Many details of the molecular and cellular mechanisms involved in *Mycobacterium bovis* bacillus Calmette-Guérin (BCG) immunotherapy of bladder cancer have been discovered in the past decades. However, information on a potential role for macrophage cytotoxicity as an effector mechanism is limited. Macrophages play pivotal roles in the host innate immunity and serve as a first line of defense in mycobacterial infection. In addition to their function as professional antigen-presenting cells, the tumoricidal activity of macrophages has also been studied with considerable interest. Studies have shown that activated macrophages are potent in killing malignant cells of various tissue origins. This review summarizes the current understanding of the BCG-induced macrophage cytotoxicity toward bladder cancer cells with an intention to inspire investigation on this important but underdeveloped research field.

## 1. Role of Macrophages in BCG Immunotherapy of Bladder Cancer

Intravesical instillation of BCG is used for the treatment of superficial transitional cell carcinoma (TCC) of the bladder to reduce recurrence and progression of this disease. Since its first therapeutic application in 1976 [1], major efforts have been directed to decipher the mechanisms through which BCG mediates antibladder cancer immunity. Although the exact mechanism of BCG action remains elusive, an induction of localized cellular immune responses reflecting activation of various types of immune cells has been proposed [2, 3]. Potential effector cells responsible for tumor killing include MHC-restricted cytotoxic T cells (CD8^+^ CTL) and CD4^+^ T cells [4, 5], MHC-nonrestricted cells such as natural killer (NK) cells [6–8], lymphokine-activated killer (LAK) cells [8, 9] and BCG-activated killer (BAK) cells [10–12], CD1-restricted CD8^+^ T cells [13], *γ* *δ* T cells [14–16], natural killer T (NKT) cells [15–17], neutrophils [18, 19], and macrophages [20–22]. Several lines of evidence suggest that macrophages actively mediate antibladder cancer immunity induced by BCG. Following BCG instillation, increased numbers of macrophages, along with T cells, NK cells, dendritic cells (DC), and neutrophils, were observed in bladder cancer infiltrates and the peritumoral bladder wall [2, 3, 23–26]. The voided urine of bladder cancer patients after BCG treatment also contained increased numbers of macrophages and other types of immune cells [27–29]. Moreover, a transient secretion of various cytokines and chemokines in patients' urine after intravesical BCG treatment has been reported, including those predominantly produced by macrophages such as TNF-*α*, IL-6, IL-10, IL-12, and IL-18 [2, 3, 29–35]. Furthermore, both human and murine macrophages have been observed to produce TNF-*α*, IFN-*γ*, IL-6, IL-10, IL-12, and IL-18 in response to BCG stimulation *in vitro* [36–39]. In addition to their cytokine/chemokine production, both human and murine macrophages can also act as cytotoxic effector cells toward bladder cancer cells upon activation by BCG *in vitro* [40–46]. It has been postulated that, in the case of intravesical BCG treatment of bladder cancer, the bladder mucosa develops BCG-mediated inflammatory responses, which involve the initial binding of BCG to fibronectin on the urothelium [47], followed by internalization of BCG by urothelial cells, and subsequent activation of various immune cells including macrophages in the bladder wall [47, 48]. Once activated by BCG, macrophages gain effector functions to act as inflammatory, microbicidal, and tumoricidal cells in BCG-induced anti-bladder cancer immune responses.

## 2. BCG-Induced Macrophage Cytotoxicity

The BCG induction of macrophage cytotoxicity can be demonstrated *in vitro* through a cytotoxicity assay in which macrophages are preactivated with BCG and then coincubated with radioisotope-labeled bladder cancer cells, followed by analysis of radioisotope release from target cells in culture supernatants. Using this method, Pryor and associates reported that BCG could enhance monocytes/macrophages from human peripheral blood to kill bladder cancer UCRU-BL-17 cells [40]. Although naïve monocytes/macrophages exhibited minimum cytotoxicity, the cytotoxic activity of the cells was significantly enhanced upon BCG stimulation. In addition, BCG appeared to be superior to IFN-*α*, IFN-*γ*, and IL-2 in enhancing monocyte/macrophage-mediated cytotoxicity. However, the BCG-activated monocytes/macrophages failed to show consistent cytotoxicity toward bladder cancer 5637, T24, and J82 cells, although all these cell lines including UCRU-BL-17 were similarly susceptible to BCG direct killing [40]. Possible explanations for this discrepancy might relate to differential expression of the receptors of cytotoxic effector molecules on target cells or adhesion molecules on both effector and target cells. These possibilities wait to be investigated.

Yamada and associates reported that thioglycollate-elicited peritoneal macrophages from C3H/HeN mice were cytotoxic to syngeneic bladder cancer MBT-2 cells upon BCG stimulation [41–43]. This BCG-induced macrophage cytotoxicity was correlated with the production of TNF-*α*, IFN-*γ*, and IL-12 by macrophages. In addition, viable BCG was observed to be superior to nonviable BCG for the induction of macrophage cytotoxic activity and cytokine production [42, 43]. Yamada and associates further observed that C3H/HeN macrophages produced prostaglandin (PG) E_2_ along with other cytokines in response to BCG stimulation [43]. PGE_2_ exhibited an inhibitory effect on BCG-induced C3H/HeN macrophage cytotoxicity toward MBT-2 cells [43]. Inhibition of cyclooxygenase (COX)-1 and/or COX-2, the enzymes responsible for the formation of prostanoids such as PGE_2_ [49], during BCG stimulation could enhance the macrophage cytotoxic activity and production of TNF-*α* and IFN-*γ* [43]. These observations suggested that an inhibitor of PG synthesis might be potentially useful for enhancing BCG immunotherapy of bladder cancer. Subsequently, our own studies demonstrated the ability of BCG to induce the cytotoxic activity of C3H/HeN macrophages toward MBT-2 cells [44–46]. We were able to show that BCG was superior to lipopolysaccharide (LPS) for the induction of macrophage cytotoxicity [45, 46]. Our investigations further demonstrated that endogenous T helper type (Th) 1 cytokines IFN-*γ*, IL-12, and IL-18 as well as proinflammatory cytokine TNF-*α* played an important role in BCG-induced C3H/HeN macrophage cytotoxicity and that supplementation of BCG with exogenous recombinant (r) IL-12 and rIL-18 could enhance the macrophage cytotoxicity [45].

In addition to C3H/HeN macrophages, we have also observed that BCG could induce cytotoxic activity of macrophages from C57BL/6 mice toward syngeneic bladder cancer MB49 cells [46]. Further, both BCG-activated C3H/HeN macrophages and BCG-activated C57BL/6 macrophages were capable of killing allogeneic bladder cancer cells reciprocally, although such killing was less potent than those toward syngeneic bladder cancer cells [46]. Compared to BCG-activated C3H/HeN macrophages, the cytotoxic activity of BCG-activated C57BL/6 macrophages was substantially weak. Since both MBT-2 cells and MB49 cells showed similar susceptibility to macrophage-derived cytotoxic effector molecules such as TNF-*α* and nitric oxide (NO) [46], Th2 cytokine(s) produced by BCG-activated macrophages might be causative for the reduced cytotoxicity of C57BL/6 macrophages. To determine this, we conducted a study in which macrophages of both genetic backgrounds were assessed in parallel for their cytokine and NO production [46]. We found that macrophage-derived IL-10 played an inhibitory role in BCG-induced macrophage cytotoxicity and was responsible for the BCG induction of reduced cytotoxic activity of C57BL/6 macrophages [46]. However, differential expression of IL-10 receptors on bladder cancer cells and/or macrophages from the two different genetic backgrounds might also play a role in susceptibility of the tumor cells to BCG-induced macrophage cytotoxicity. Thus, although both MBT-2 cell and MB49 cell-derived bladder cancers in syngeneic mice, two widely used bladder cancer models, have been demonstrated to be similarly responsive to intravesical BCG treatment [50, 51], the detailed mechanisms through which BCG induces antitumor immunity in these two distinctive genetic backgrounds of mice are apparently not identical.

## 3. Mechanism of BCG-Induced Macrophage Cytotoxicity

Multiple effector mechanisms are involved in the killing of bladder cancer cells by BCG-induced macrophage cytotoxicity. Both direct effector-target cell contact and release of soluble cytotoxic factors (such as TNF-*α*,IFN-*γ* and NO) are required for effective killing of bladder cancer cells by BCG-activated macrophages [45]. The effector-target cell contact was observed to contribute to approximately 50% of the total killing of MBT-2 cells by BCG-activated C3H/HeN macrophages [45]. However, knowledge about the mechanism of cell-cell contact mediated killing is scarce. BCG infection may result in up-regulated expression of adhesion molecules such as lymphocyte function-associated antigen-1 (LFA-1) or apoptosis-inducing molecules such as Fas ligand and TRAIL on macrophages [52–54]. Acquirement of these surface molecules could direct macrophages to bind to and kill bladder cancer cells. This assumption of phenotypical changes of macrophages has been a subject of study that should yield valuable insights into the mechanism of BCG action. 

Soluble factors released from macrophages were also found to account for approximately 50% of the total killing of MBT-2 cells by BCG-activated C3H/HeN macrophages [45]. Soluble factors released from BCG-activated human monocytes/macrophages contributed to the total killing of UCRU-BL-17 cells even higher than effector-target cell contact [40]. Production of TNF-*α*, IFN-*γ*, and NO by macrophages in response to BCG stimulation has been observed *in vitro* [36–39, 41–46, 55, 56]. These effector factors are known to be cytotoxic to bladder cancer cells [45, 46, 57–59] and kill target cells through the apoptotic and/or necrotic pathways [60–62]. In addition to the killing of bladder cancer cells, both TNF-*α* and IFN-*γ* are also known to work through autocrine for macrophage activation and NO production [38, 56, 63–65]. NO is a reactive nitrogen/oxygen intermediate with strong cytotoxicity toward malignant cells. It has been demonstrated to kill both human and murine bladder cancer cells *in vitro* [46, 66–68]. Thus, production of TNF-*α*, IFN-*γ*, and NO renders macrophages activation and cytotoxic activity. Indeed, high BCG-induced macrophage cytotoxicity has been observed to correlate with high production of these cytotoxic effector molecules [45, 46].

## 4. Role of Th1 and Th2 Cytokines in BCG-Induced Macrophage Cytotoxicity

Macrophages produce inflammatory cytokines, such as TNF-*α*, IFN-*γ*, IL-6, IL-10, IL-12, and IL-18, in response to BCG stimulation [36–39, 41–46]. These cytokines act in a reciprocal fashion and are tightly controlled for a proper induction of Th1/Th2 immune responses. Acquirement of a Th1 immune response is essential to effective BCG immunotherapy of bladder cancer [2, 3, 32–34, 69]. In addition, Th1 cytokines are also found to be vital for the BCG induction of macrophage tumoricidal activity. 

### 4.1. Role of Th1 Cytokines

It has been observed that Th1 cytokines IFN-*γ*, IL-12, and IL-18 play a positive role in BCG-induced macrophage cytotoxicity toward bladder cancer cells [43, 45]. Proinflammatory cytokine TNF-*α* has also been observed to be critical in the BCG-induced macrophage cytotoxicity [43, 45]. Blockage of these endogenous cytokines by neutralizing antibodies significantly reduced BCG-induced C3H/HeN macrophage cytotoxicity toward MBT-2 cells [43, 45]. Among these cytokines, neutralization of TNF-*α* led to the most severe inhibition on macrophage cytotoxicity [45]. To support this role of Th1 cytokines, it has been observed that supplementation of BCG with exogenous rIL-12 or rIL-18 resulted in an increase in the induction of macrophage cytotoxicity [45]. Moreover, C3H/HeN macrophages treated with rBCG expressing IL-18 exhibited increased killing of MBT-2 cells, along with increased production of TNF-*α* and IFN-*γ* [44, 45]. In addition, C3H/HeN macrophages developed enhanced cytotoxicity after stimulation with BCG plus rIL-2 or with rBCG expressing IL-2 [41, 45]. These observations suggest that Th1 cytokines play a pivotal role in BCG-induced macrophage cytotoxicity and that combination of BCG with Th1 cytokines may enhance the effect of BCG in the treatment of bladder cancer patients.

### 4.2. Role of Th2 Cytokines

IL-10, one of the major Th2 cytokines produced by macrophages, is inhibitory to the BCG induction of macrophage cytotoxicity. High IL-10 production was observed to correlate with low killing of MB49 cells and reduced production of TNF-*α*, IL-6, and NO by BCG-activated C57BL/6 macrophage [46]. To support this inhibitory role of IL-10, neutralizing endogenous IL-10 during BCG stimulation resulted in increased BCG-induced C57BL/6 macrophage cytotoxicity toward MB49 cells, along with increased production of TNF-*α* and NO [46]. Since supplementation of exogenous rTNF-*α* failed to enhance the BCG-induced macrophage cytotoxicity, IL-10 appeared to play a predominant role in controlling the induction of C57BL/6 macrophage cytotoxicity by BCG [46]. Further evidence supporting the inhibitory role of IL-10 includes the studies that showed high BCG-induced cytotoxicity toward MB49 cells by macrophages from genetically modified C57BL/6 mice lacking IL-10 (IL-10^−/−^) and reduced BCG-induced cytotoxicity toward MBT-2 cells as well as MB49 cells by C3H/HeN macrophages pretreated with exogenous rIL-10 [46]. In addition, studies have also shown that IL-10 could inhibit macrophage release of cytokines (e.g., TNF-*α*) and reactive nitrogen/oxygen intermediates (e.g., NO) [70–73]. Thus, IL-10 is inhibitory to the inflammatory, microbicidal and tumoricidal activities of macrophages. These observations suggest that blockage of IL-10 may potentially enhance the effect of BCG in the treatment of bladder cancer patients, particularly for BCG nonresponders who often develop high IL-10 levels during BCG treatment [32–34].

The role of IL-6 in BCG-induced macrophage cytotoxicity remains elusive. Production of IL-6, along with TNF-*α*, IFN-*γ*, and NO, was observed to correlate with the BCG induction of cytotoxic activity of both C3H/HeN macrophages and C57BL/6 macrophages [45, 46]. IL-6 is known to be involved in macrophage maturation [74], and thus may contribute to BCG-induced macrophage cytotxicity through its influence on macrophage release of cytotoxic effector molecules and/or expression of surface adhesion and apoptosis-inducing molecules. In addition, the fates of supplementation of exogenous rIL-6 or blockage of endogenous IL-6 on BCG induction of macrophage cytotoxicity are unknown.

## 5. Conclusion and Future View

*In vitro* studies have demonstrated the ability of BCG to induce macrophage cytotoxicity toward bladder cancer cells in both human and murine. This macrophage-mediated killing of bladder cancer cells depends on both direct effector-target cell contact and release of soluble cytotoxic factors, such as TNF-*α*,IFN-*γ*, and NO, from macrophages. Th1 cytokines IFN-*γ*, IL-12, and IL-18 as well as proinflammatory cytokine TNF-*α* play positive roles in BCG-induced macrophage cytotoxicity whereas Th2 cytokine IL-10 plays a negative role in BCG-induced macrophage cytotoxicity. Supplementation of exogenous Th1 cytokines such as rIL-12 and rIL-18 or inhibition of endogenous Th2 cytokine IL-10 enhances BCG-induced macrophage cytotoxicity. However, despite these findings, there are some unsolved issues with respect to the BCG-induced macrophage cytotoxicity: (1) how potent is the cytotoxic activity of macrophages relative to T cells, NK cells, LAK cells, BAK cells, and NKT cells in killing bladder cancer cells?, (2) what are the phenotypical changes of macrophages in response to BCG stimulation that facilitate macrophages to contact bladder cancer cells?, (3) why do BCG-activated macrophages exhibit no cytotoxicity toward some human bladder cancer cell lines?, and (4) what is the actual role of IL-6 in BCG-induced macrophage cytotoxicity toward bladder cancer cells? In addition, although BCG-induced macrophage cytotoxicity has been demonstrated *in vitro*, there is a lack of evidence supporting the role of macrophage cytotoxicity in BCG-mediated eradication of bladder cancer *in vivo*. As technology develops, we anticipate that these issues will be approached and answered. A better understanding of the role of macrophages in BCG mediated immune responses will no doubt add to our proper design and application of BCG immunotherapy for bladder cancer.
